# Genome-Wide Resequencing Identification of Loci Associated with Early Maturity in Upland Cotton and Functional Validation of *GhGATA9*

**DOI:** 10.3390/plants15213287

**Published:** 2026-10-25

**Authors:** Yuli Lu, Luan Sun, Penghui Yi, Ning Zhang, Qiming Wang, Xiaoju Tu

**Affiliations:** 1 College of Agronomy, Hunan Agricultural University, Changsha 410128, China; 2 Yuelushan Laboratory, Changsha 410128, China; 3 College of Bioscience and Biotechnology, Hunan Agricultural University, Changsha 410128, China

**Keywords:** upland cotton, early maturity, whole-genome resequencing, selective sweep analysis, *GhGATA9* gene

## Abstract

Early maturity is an important agronomic trait in upland cotton (*Gossypium hirsutum* L.), contributing to an increased cropping index, avoidance of extreme weather, and facilitation of mechanized cotton production. In this study, 15 early-maturing and 15 late-maturing upland cotton accessions were subjected to whole-genome resequencing to identify genomic regions and key genes associated with early maturity. A total of 2,684,227 high-quality single-nucleotide polymorphisms (SNPs) were identified. Based on the integrated Fst–θπ analysis, 16 genomic regions under selection in the early-maturing group were identified, encompassing 80 selected genes. Functional enrichment analysis showed that these genes were mainly involved in cellular membrane organization, fatty acid metabolism, and biotin metabolism. By further examining genes associated with plant growth and development, flowering regulation, and transcriptional regulation, a key transcription factor gene, *Gossypium hirsutum* GATA transcription factor 9 (*GhGATA9*), was identified as a candidate gene associated with early maturity. Virus-induced gene silencing (VIGS) assays showed that silencing *GhGATA9* delayed both budding and flowering by 2.4 days, indicating that *GhGATA9* may function as a positive regulator of early maturity in upland cotton. These findings provide novel molecular markers and a candidate gene for the molecular breeding of early-maturing cotton varieties.

## 1. Introduction

Cotton (*Gossypium hirsutum* L.) is an important cash crop that provides natural fibers, edible oil, and plant proteins, and plays a vital role in the textile and chemical industries [1]. However, with the intensification of global climate change, the increasing frequency of extreme weather events poses a serious threat to crop growth and yield stability [2]. Shortening the crop growth cycle is considered an effective strategy for mitigating the adverse effects of abnormal climatic conditions [3]. In cotton, in particular, the development of early-maturing cultivars can not only reduce yield losses caused by extreme weather events during spring and autumn but also facilitate double-cropping systems, thereby substantially improving land-use efficiency [4]. The formation of early maturity in cotton is coordinately regulated by multiple factors and is characterized by several developmental traits, including squaring time, flowering time, the node position of the first fruiting branch, and boll opening rate [5]. These traits are primarily determined by genetic background but are also highly responsive to environmental conditions and cultivation practices.

In recent years, rapid advances in molecular biology and genomics have provided new perspectives for elucidating the mechanisms underlying crop earliness. Whole-genome sequencing (WGS) is a high-throughput sequencing technology that enables comprehensive characterization of an organism’s genome, including both coding and non-coding regions [6]. Compared with conventional molecular marker-based approaches, WGS enables genome-wide detection of single-nucleotide polymorphisms (SNPs), structural variants (SVs), and copy number variations (CNVs), and is particularly effective for identifying functional variants in non-coding regulatory regions [7]. By performing whole-genome resequencing of 436 upland cotton germplasm accessions, previous researchers constructed a variation map containing 10.11 million high-quality SNPs and identified two genetic hotspots associated with earliness on chromosomes D03 and A05 through association analysis. Among these SNPs, the marker rsD03_39122594 was significantly associated with a shortened growth period. Further investigation revealed that *Ghir_D03G011310* in this region was subjected to strong artificial selection during the breeding of early-maturing cotton, and loss of its function resulted in delayed squaring [8]. Similarly, in rice, WGS combined with a genome-wide association study (GWAS) identified the *Ef-cd* gene, which promotes early maturity without yield penalties by enhancing nitrogen metabolism and photosynthetic efficiency [9].

WGS also enables the precise detection of large-scale structural variants, such as chromosomal inversions and translocations, which are frequently associated with key agronomic traits. In foxtail millet, an ultra-early-maturing mutant, “Xiaomi,” was generated through EMS mutagenesis. Genome sequencing revealed a 2.1-Mb inversion on chromosome Dt7, which reduced the expression of the photoperiod-responsive gene *SiPRR37*, thereby shortening the growth period to 60 days [10]. In addition, WGS overcomes the limitations of studies focused primarily on coding regions by enabling the identification of genetic effects associated with regulatory elements such as promoters and enhancers. In the early-maturing cotton line RIL182, previous studies reported that the methylation level of the *GhAPL* promoter was 42% lower than that in the late-maturing cultivar G2005. Targeted demethylation of this region using CRISPR/dCas9-TET1 increased *GhAPL* expression by 3.5-fold and advanced the squaring stage by 6 days. Further analysis revealed that an SNP cluster (chr08:7,654,322–7,656,891) in this region overlapped with the binding site of the DNA demethylase *GhDME*, suggesting the formation of an epigenetic regulatory module [11].

Moreover, integrative multi-omics analysis based on WGS data has become an important strategy for elucidating gene functions. By integrating genomic and transcriptomic data, previous studies identified *GhGRAS55* as a gene specifically highly expressed in early-maturing cotton. Silencing of *GhGRAS55* reduced the activities of key enzymes involved in the phenylpropanoid metabolic pathway, ultimately delaying flowering by 9.3 days [12]. Although numerous studies have investigated genes associated with early maturity in cotton using whole-genome sequencing approaches [13,14,15], a systematic understanding of the genetic variation patterns among early- and late-maturing cotton materials and the key regulatory genes underlying these differences remains limited.

Therefore, in this study, we performed whole-genome resequencing of 30 upland cotton germplasm accessions, comprising 15 early-maturing and 15 late-maturing cultivars, which were selected in our previous screening. Genome-wide SNPs and insertion–deletion (InDel) variants were comprehensively identified. Candidate genes and genetic variants associated with early maturity were subsequently screened through functional annotation and enrichment analyses, while protein structure prediction and evolutionary conservation analyses were employed to further investigate their potential biological significance. The preliminary functional validation of the key candidate gene was further validated using quantitative real-time polymerase chain reaction (qRT-PCR) and virus-induced gene silencing (VIGS). These findings provide new insights into the genetic basis underlying early maturity in cotton.

## 2. Results

### 2.1. Field Growth Duration of Early- and Late-Maturing Cotton Materials

**Figure 1:**
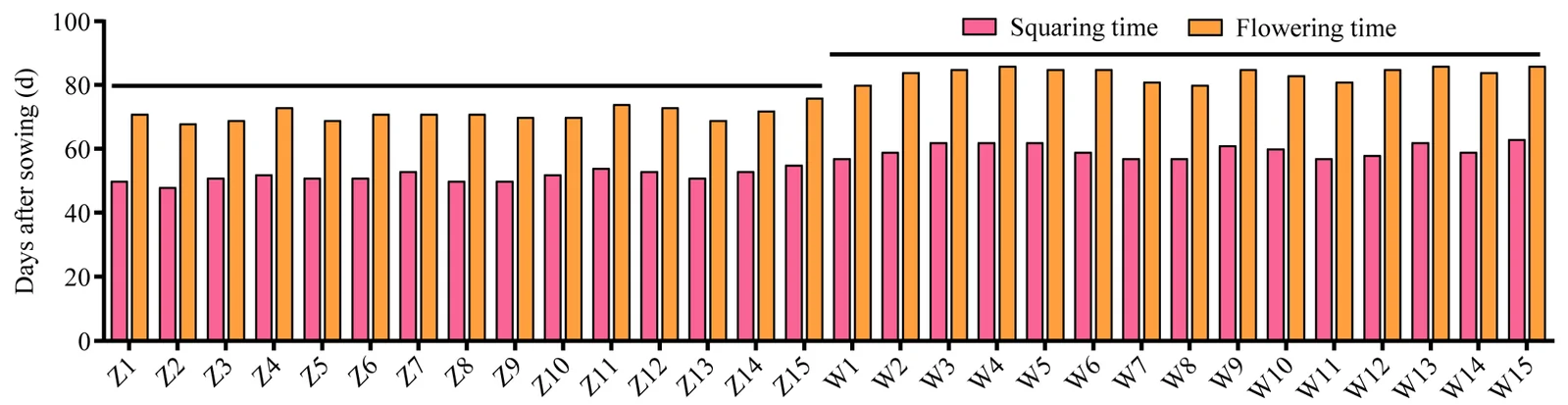
Flowering and squaring time of 15 early-maturing cotton varieties and 15 late-maturing cotton varieties. Z1-Z15 and W1-W15 represent 15 early-maturing and 15 late-maturing cotton accessions, respectively.

Field investigations of the developmental progression of early- and late-maturing cotton materials revealed significant differences in squaring and flowering times between the two groups (Figure 1). Compared with early-maturing cotton materials, late-maturing materials exhibited a delay of 8.0 days in average squaring time and 12.6 days in average flowering time, respectively. Among the tested materials, Z2 showed the shortest time to squaring and flowering, requiring 48 and 68 days, respectively, whereas W15 exhibited the longest developmental duration, with squaring and flowering occurring at 63 and 86 days, respectively.

### 2.2. Quality Control and Alignment of Whole-Genome Resequencing Data

Whole-genome resequencing was performed on 15 early-maturing and 15 late-maturing cotton accessions. After filtering low-quality reads, a total of 774 Gb of clean data was obtained. Quality control showed that the Q20 values of all samples exceeded 96.80%, the Q30 values exceeded 91.38%, and the GC content ranged from 34.23% to 35.30%. The clean reads were then aligned to the reference genome, yielding an average mapping rate of 99.08%. The 1× genome coverage (≥1× depth) exceeded 98.84%, and the 4× coverage (≥4× depth) exceeded 90.25% (Table S1), indicating that the sequencing data were of high quality and uniformly distributed, and therefore suitable for downstream analyses.

### 2.3. SNP Quality Control Analysis

A total of 2,684,227 high-quality SNPs were identified through resequencing. Intergenic SNPs accounted for the largest proportion (2,360,509; 87.9%), indicating that most variations were concentrated in non-coding regulatory regions. A total of 122,059 intronic SNPs (4.5%) were identified, which may be associated with splicing regulation or post-transcriptional modification. A total of 55,703 exonic SNPs (2.1%) were detected, among which non-synonymous SNPs were the most common (34,440; 61.8%) and were likely to affect protein function. Synonymous SNPs accounted for 20,377 (36.6%). In addition, 671 stop-gain (1.2%) and 215 stop-loss (0.4%) SNPs were observed, suggesting that some gene functions might have been completely lost or abnormally extended. A total of 222 splicing SNPs were identified, which require further validation to determine their impact on mRNA splicing efficiency. In total, 1,852,021 transition (ts) SNPs and 832,206 transversion (tv) SNPs were identified, yielding a ts/tv ratio of 2.225 (Table 1). This ratio fell within the expected range for cotton genome variation (2.0–3.5), indicating reliable data quality [16]. The proportion of transitions (69.0%) was significantly higher than that of transversions (31.0%), consistent with the purine–pyrimidine substitution bias in eukaryotic genomes [17].

### 2.4. Population Genetic Relationship Analysis of Early- and Late-Maturing Cotton Materials

**Figure 2:**
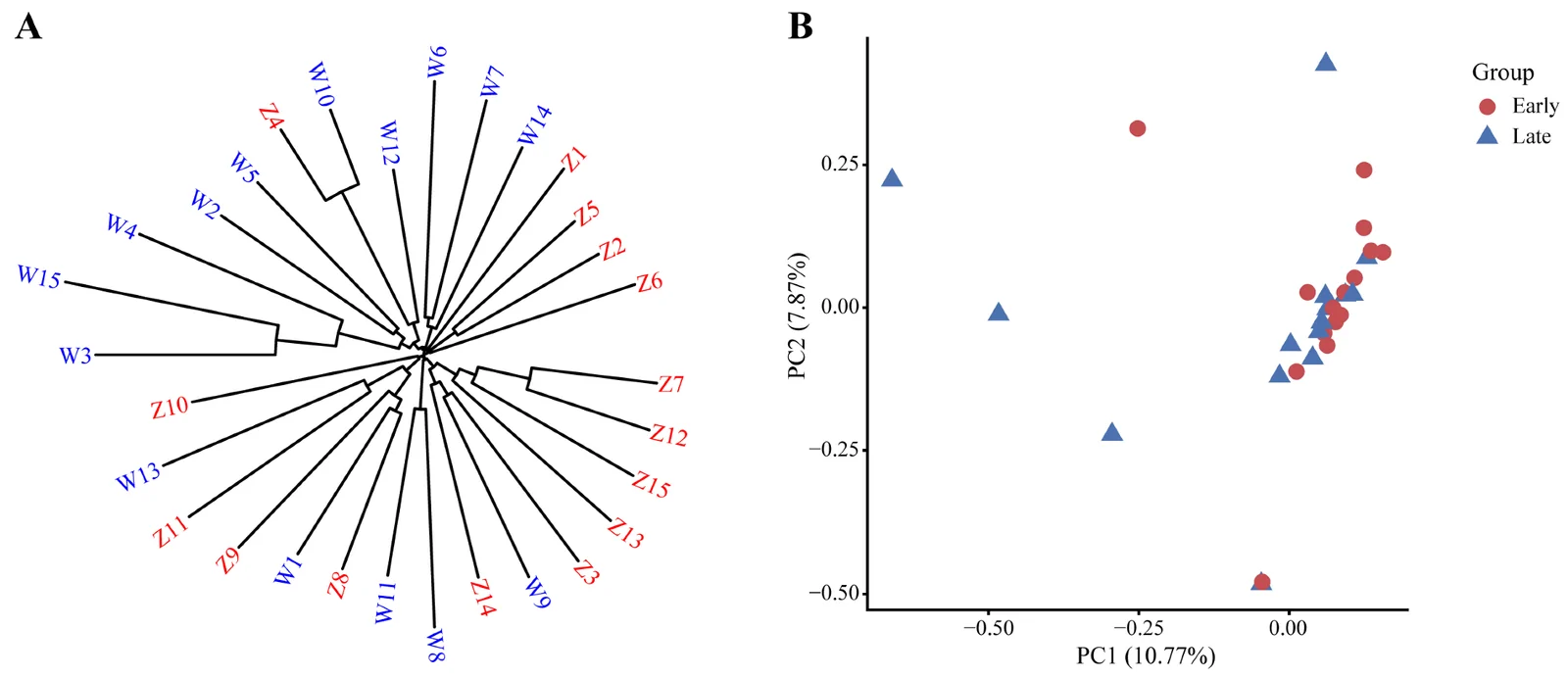
Phylogenetic relationship and principal component analysis of early- and late-maturing cotton materials based on genome-wide SNP markers. (**A**) Phylogenetic tree. (**B**) Principal component analysis (PCA).

A total of 2,684,227 high-quality SNPs were used to construct a phylogenetic tree and perform principal component analysis (PCA) for the 30 cotton materials (Figure 2). The phylogenetic tree analysis revealed that early-maturing (Z) and late-maturing (W) cotton materials did not form two completely distinct evolutionary branches based on their maturity phenotypes. Instead, individuals from the two groups were interspersed throughout the phylogenetic tree, and several early-maturing materials clustered with late-maturing materials within the same branches. These results suggested that the two groups shared a similar genetic background and might have experienced genetic exchange during their breeding history. PCA showed that PC1 and PC2 explained 10.77% and 7.37% of the total genetic variation, respectively. Most early- and late-maturing materials exhibited overlapping distributions, without forming clearly separated genetic clusters, although a small number of individuals showed distinct separation patterns. The PCA results were consistent with the phylogenetic tree analysis, further indicating that early- and late-maturing cotton materials possessed a complex genetic relationship rather than forming two genetically isolated groups.

### 2.5. Select Elimination Analysis

**Figure 3:**
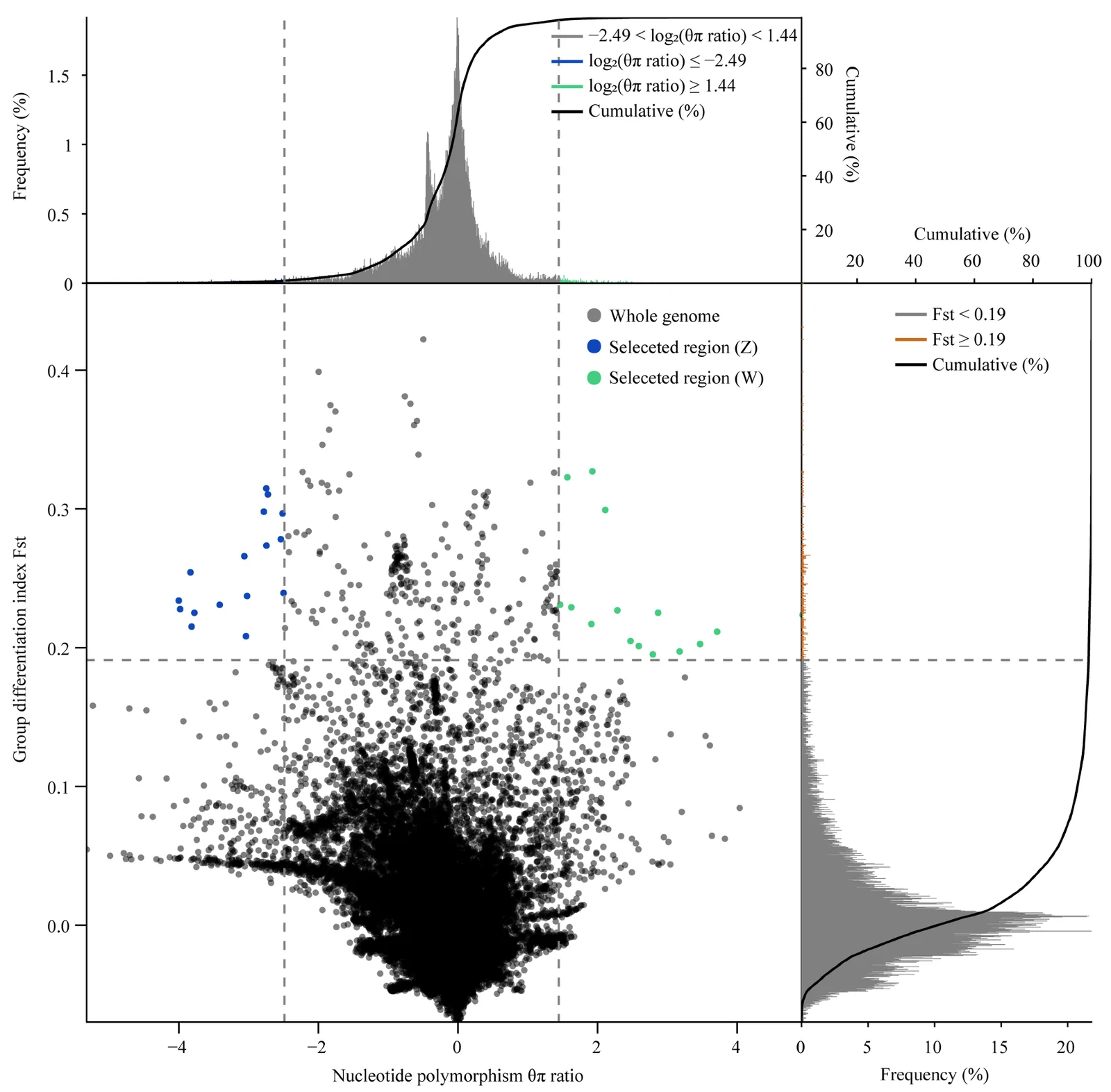
Selection signal region identification map of upland cotton. The *x*-axis represents the log_2_ θ_π_ ratio, and the *y*-axis represents the F_st_ value. The top and right panels show the frequency distributions of the θ_π_ ratio and F_st_, respectively, while the central scatter plot displays the corresponding F_st_ and θ_π_ ratio values across different genomic windows. The blue and green shaded areas indicate the top 5% regions identified based on the θ_π_ ratio, and the red shaded area represents the top 5% regions identified based on F_st_.

The Fst–θπ joint analysis was performed to identify genomic regions under selection by integrating signals from Fst and the π ratio. As shown in Figure 3, the blue and green regions indicate genomic regions under selection in the early-maturing (Z) and late-maturing (W) groups, respectively, based on the top 1% thresholds of Fst and the π ratio. A total of 16 genomic regions under selection were detected in the early-maturing group, encompassing 80 selected genes (Table S2).

### 2.6. Functional Enrichment Analysis of Selected Genes

**Figure 4:**
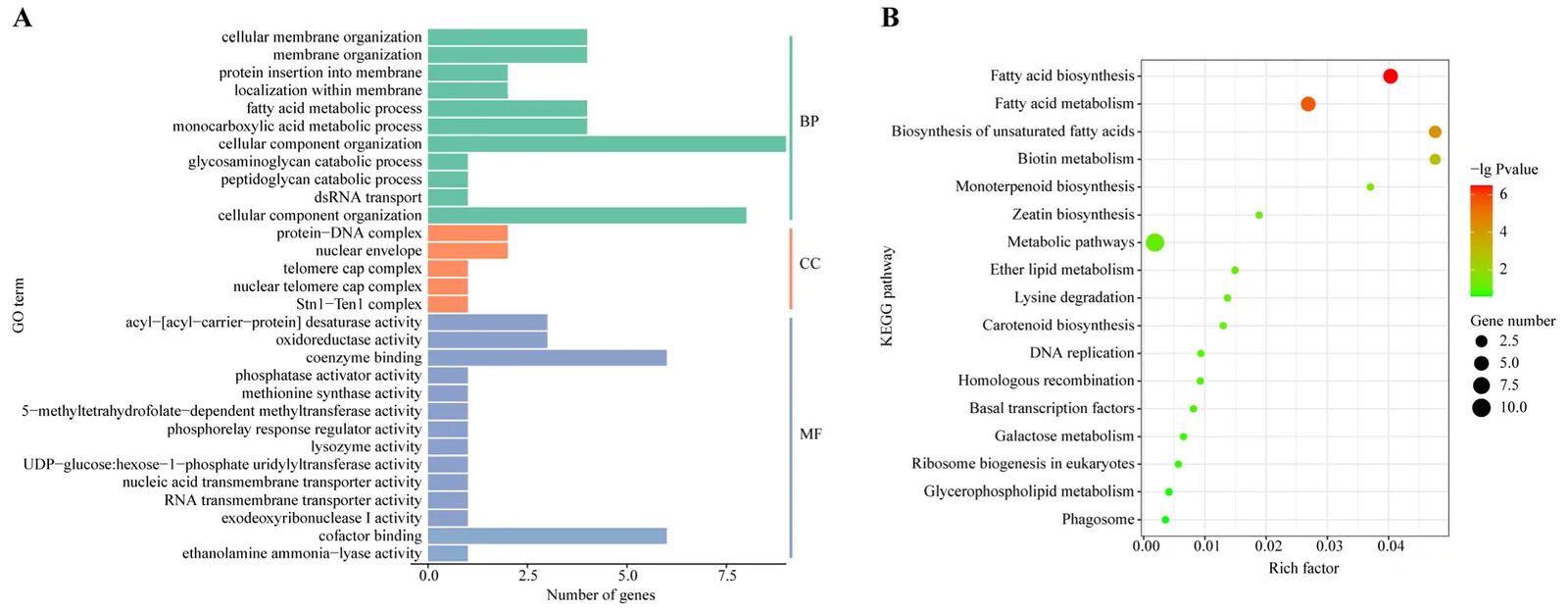
GO (**A**) and KEGG (**B**) enrichment analyses of candidate genes.

To further investigate the biological functions and metabolic pathways associated with the selected genes in the early-maturing group, GO and KEGG enrichment analyses were performed. The GO enrichment analysis showed that, among biological process (BP) terms, the most significantly enriched terms were cellular membrane organization, membrane organization, and protein insertion into membrane. For cellular component (CC), protein–DNA complex and nuclear membrane were the most significantly enriched terms. In the molecular function (MF) category, acyl-[acyl carrier protein] desaturase activity, oxidoreductase activity, and coenzyme binding were among the most significantly enriched terms (Figure 4A). KEGG enrichment analysis revealed that fatty acid biosynthesis, fatty acid metabolism, and biotin metabolism were the most significantly enriched pathways. In addition, unsaturated fatty acid biosynthesis and monoterpenoid biosynthesis were also significantly enriched (Figure 4B).

### 2.7. Bioinformatics Analysis of Candidate Genes

To further explore key candidate genes associated with early maturity in cotton, the 80 candidate genes were evaluated based on multiple criteria, including (i) functional annotation related to plant growth, development, flowering regulation, or transcriptional regulation; (ii) transcription-factor properties; (iii) previous functional studies in plants; and (iv) the presence of genetic variants within the candidate genes or their flanking regions (Table S2). Among these candidates, *GH_A11G2894* was prioritized because it encodes a GATA-type transcription factor, a class of regulators known to participate in plant developmental processes and flowering-regulation pathways. Notably, one significant SNP located in the upstream flanking region of *GhGATA9* (A11:108163990, T > C) was identified from our re-sequencing dataset. This variant exhibited a striking allelic-frequency divergence between early- and late-maturing accessions: the frequency of the alternative C allele reached 56.7% in early-maturing materials but only 7.1% in late-maturing materials. Such strong population-level differentiation implies that *GhGATA9* may have undergone positive selection during the phenotypic differentiation between early- and late-maturing cotton germplasm. Therefore, *GH_A11G2894* (*GhGATA9*) was selected as a high-confidence promising candidate gene for further functional validation.

#### 2.7.1. Structural and Physicochemical Characterization of the GhGATA9 Protein

The physicochemical properties and structural characteristics of the GhGATA9 protein were analyzed using online bioinformatics tools, including ProtScale, ProtParam, NetPhos 3.1, SignalP 5.0, TMHMM 2.0, and SOPMA (Figures S1–S3). The results showed that the GhGATA9 protein exhibited hydrophilic characteristics, with a grand average of hydropathicity (GRAVY) value of −0.542. A total of 41 potential phosphorylation sites were predicted, including 23 serine (Ser), 15 threonine (Thr), and 3 tyrosine (Tyr) residues. No typical signal peptide or transmembrane helix was detected, suggesting that GhGATA9 is a non-secretory and non-transmembrane protein. Secondary structure prediction indicated that GhGATA9 mainly consisted of random coils (88.66%), followed by α-helices (8.59%) and extended strands (2.75%), with no β-turn structure detected. These results provide basic structural information for further functional characterization of *GhGATA9*.

#### 2.7.2. Protein–Protein Interaction Network Analysis of the Arabidopsis Homolog of GhGATA9

To further investigate the potential function of the GhGATA9 protein, a protein–protein interaction network analysis was performed based on its Arabidopsis homolog (Figure S4). The results showed that GATA9 interacted with GATA24, ARF30, BHLH118, ANL2, FEZ, and AIL6. These proteins are mainly involved in biological processes such as transcriptional regulation, hormone responses, and floral organ development, suggesting that GATA9 may affect reproductive growth in cotton by participating in plant developmental regulatory networks.

#### 2.7.3. Expression of GhGATA9 in Different Cotton Tissues

To investigate the spatiotemporal expression pattern of GhGATA9, publicly available transcriptome data from the upland cotton standard line TM-1 were obtained from the CottonFGD database (http://cotton.zju.edu.cn/download.html, accessed on 10 July 2026) and used to analyze the expression profile of GhGATA9 in different tissues (Figure S5). The results showed that *GhGATA9* exhibited the highest expression levels in the bracteole, leaves, stamens, and stems, followed by the pistils, receptacles, and roots, whereas its expression level was lowest in the petals.

### 2.8. Functional Validation of GhGATA9

**Figure 5:**
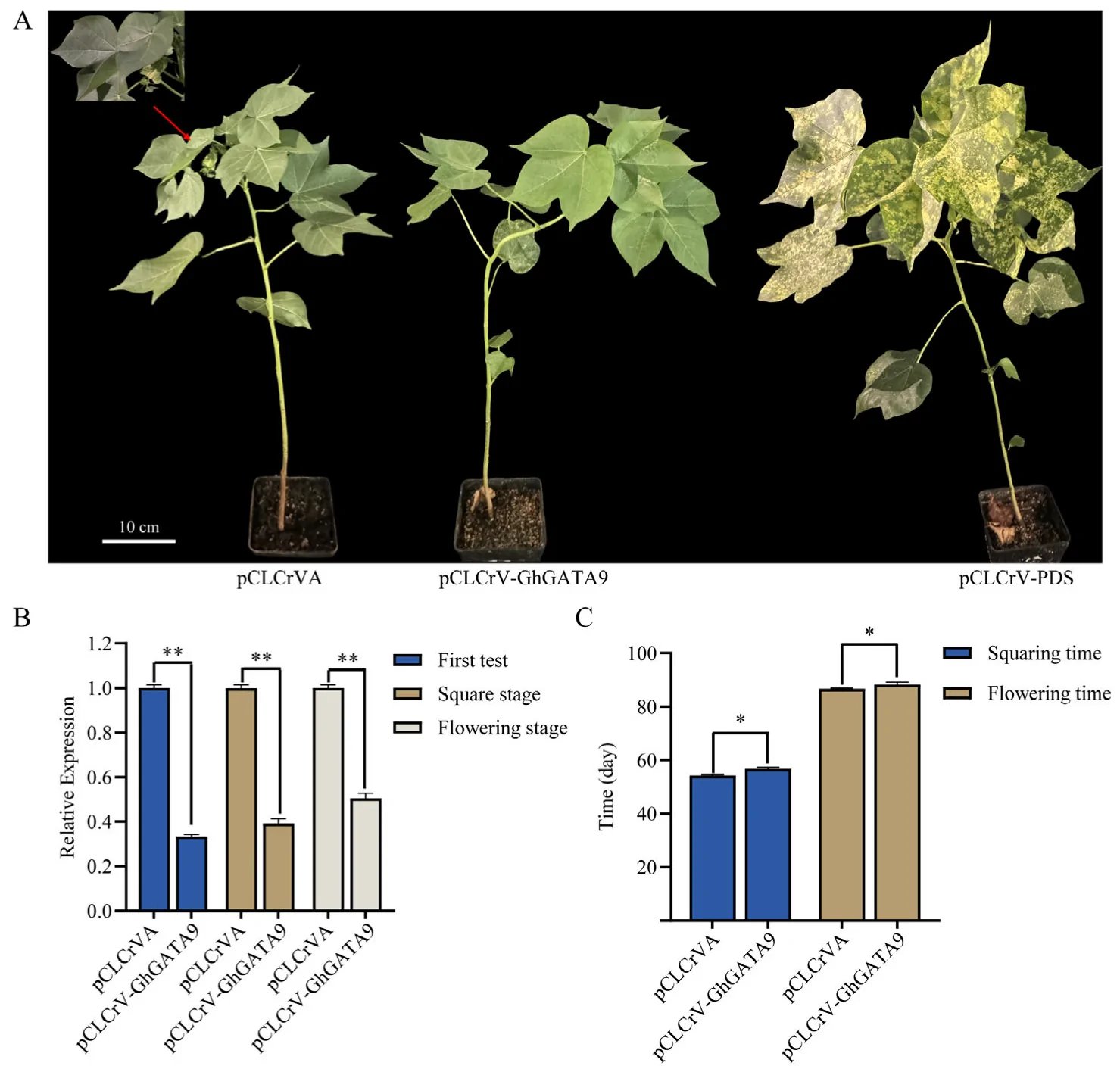
VIGS of *GhGATA9* led to changes in cotton squaring and flowering time. (**A**) Phenotype of the negative control (pCLCrVA), positive control (PDS), and gene-silenced plants after 55 days of growth. (**B**) Silencing efficiency of *GhGATA9* at three different time points. (**C**) Statistics of budding and flowering time of pCLCrVA and gene-silenced plants. Bar graphs represent mean values ± SD. The first test was performed when the positive control exhibited a chlorosis phenotype. Statistical significance was determined using one-way ANOVA followed by Tukey’s multiple comparison test; * and ** represent significant differences at the 0.05 and 0.01 levels, respectively.

To investigate the role of *GhGATA9* in regulating early maturity in upland cotton, virus-induced gene silencing (VIGS) was employed to silence *GhGATA9*. At 55 days after inoculation, plants in the negative control group (pCLCrVA) had already initiated squaring, whereas none of the pCLCrV-*GhGATA9* plants had initiated squaring (Figure 5A). Silencing efficiency was assessed by qRT-PCR at different time points. The results showed that *GhGATA9* expression levels in the silenced plants were significantly reduced by 49.52–66.57% compared with those in the negative control plants (Figure 5B). Compared with the negative control, *GhGATA9*-silenced plants exhibited a 2.4-day delay in both squaring and flowering (Figure 5C). These results indicate that *GhGATA9* may function as a positive regulator of early maturity in upland cotton.

## 3. Discussion

Early maturity is a complex quantitative trait jointly determined by multiple genes and environmental factors, and elucidating its genetic basis has long been a major focus of cotton breeding research [18]. In this study, whole-genome resequencing was performed on 15 early-maturing and 15 late-maturing upland cotton materials. Based on the integrated FST–θπ analysis, 16 significantly selected regions were identified in the early-maturing group, encompassing a total of 80 selected genes (Figure 3; Table S2). Phylogenetic analysis and principal component analysis (PCA) revealed that the early- and late-maturing materials exhibited a certain degree of genetic differentiation (Figure 2); however, their clustering patterns were not completely consistent with maturity classification. This suggests that although genetic background differences existed among the materials, the identified selective sweep regions were unlikely to be solely attributed to overall population stratification, but may also reflect genomic regions subjected to selection during the improvement of early maturity. Previous studies analyzing genetic variation and genome-wide associations in 419 upland cotton germplasms revealed that these materials could be broadly classified into early- and late-maturing groups [19], indicating substantial genetic differentiation between early- and late-maturing cotton populations across certain genomic regions. Further functional annotation of the selected genes showed significant enrichment in biological processes such as cellular membrane organization, protein insertion into membranes, and ribonucleotide catabolic processes (Figure 4A). Previous studies have shown that changes in membrane system structure may affect cellular signal transduction, substance transport, and metabolic regulation, thereby influencing plant growth and development and the progression of maturity [20]. These findings suggest that dynamic remodeling of the membrane system, accompanied by enhanced substance transport and signal transduction, may play an important role in the formation of early maturity in upland cotton. KEGG enrichment analysis (Figure 4B) showed significant enrichment of pathways involved in fatty acid biosynthesis, fatty acid metabolism, and unsaturated fatty acid biosynthesis. Fatty acids are not only important components of cellular membranes but also function as important signaling molecules involved in plant development and stress responses [20]. Previous studies have shown that unsaturated fatty acids can influence flowering and reproductive growth by regulating membrane fluidity, hormone signal transduction, and energy metabolism [21]. In addition, significant enrichment of the biotin metabolism pathway was observed in this study. Biotin is an important coenzyme involved in fatty acid biosynthesis, and changes in its metabolic activity may further affect lipid metabolism and energy supply, thereby contributing to the formation of early maturity in cotton [22]. Therefore, we speculate that membrane system remodeling and lipid metabolism-related pathways may constitute important biological bases underlying the formation of early maturity in upland cotton.

Functional validation of the candidate gene *GhGATA9* in this study provided additional evidence for its potential involvement in the regulation of early maturity. GATA transcription factors are a highly conserved class of DNA-binding proteins that are widely involved in plant growth and development, environmental responses, and metabolic regulation. In recent years, their roles in the regulation of plant earliness have gradually been elucidated, mainly involving photoperiod signal integration, hormone pathway regulation, metabolic homeostasis, and environmental adaptation. In tomato, *SlGATA6* has been identified as a key factor in maintaining auxin gradients. By regulating the expression of the auxin transport carrier *SlLAX2* and Aux/IAA proteins, *SlGATA6* inhibits organ abscission and affects the progression of reproductive development. Loss of its function disrupts auxin distribution, accelerates organ abscission, and may indirectly shorten the growth cycle [23]. In *Arabidopsis thaliana*, B-GATA family members such as *GNC* and *GNL* regulate germination, leaf development, flowering time, and senescence [24]. In addition, a study in longan showed that *DlGATA4* expression was significantly upregulated during the globular embryo stage of somatic embryogenesis. Its expression was also induced by abscisic acid (ABA) and indole-3-acetic acid (IAA), suggesting that *DlGATA4* may promote embryo maturation through hormone signaling pathways and thereby shorten the developmental period [25]. In the present study, silencing of *GhGATA9* resulted in delayed squaring and flowering, indicating that *GhGATA9* plays a potential role in regulating developmental timing and reproductive transition in cotton. However, although *GhGATA9* was identified within an early maturity-associated selective sweep region and harbored different genetic variations between early- and late-maturing materials, whether these natural variations directly contribute to maturity differences requires further investigation. In addition, although the present study identified *GhGATA9* as a promising candidate gene through selective sweep analysis, functional annotation, genetic variation screening, and VIGS validation, the possibility that other candidate genes within the selected regions also contribute to early maturity cannot be excluded. Further studies involving allele-specific functional analyses, overexpression or complementation experiments, and larger populations will be required to determine the causal relationship between *GhGATA9* variation and early maturity.

In this study, whole-genome resequencing combined with selective sweep analysis identified several genomic regions associated with early maturity in upland cotton, which were distributed on chromosomes A03, A06, A10, A11, A12, D03, D10, and D13 (Table S2). Comparison with previously reported QTLs and GWAS loci associated with early maturity revealed both consistencies and differences among studies. Notably, the selective sweep region identified on chromosome D03 was consistent with previous reports indicating that D03 represents an important genomic region controlling early maturity-related traits, including flowering time, first fruiting branch node, and growth period in upland cotton [3]. In addition, a large-scale genome-wide resequencing and GWAS study identified multiple early maturity-associated loci and showed that some GWAS signals overlapped with genomic regions under selection during cotton improvement [8]. However, most selective sweep regions identified in the present study did not completely overlap with previously reported QTL intervals, which may be explained by differences in genetic backgrounds, population structures, evaluated maturity-related traits, and analytical strategies among studies. Therefore, the genomic regions identified in this study may represent complementary genetic loci contributing to early maturity variation in upland cotton. VIGS analysis showed that silencing *GhGATA9* significantly delayed squaring and flowering, indicating that *GhGATA9* is involved in the regulation of early maturity in cotton. However, the present study did not further examine changes in the expression of key flowering-regulatory genes, such as *FT*, *SOC1*, *LFY*, and *AP1*. Therefore, the specific molecular mechanism through which *GhGATA9* regulates flowering and early maturity in cotton remains unclear. Future studies combining expression profiling, identification of downstream target genes, and molecular interaction analyses will be required to further elucidate the regulatory network of *GhGATA9*.

## 4. Materials and Methods

### 4.1. Plant Resources

The experimental materials consisted of upland cotton germplasm provided by the Cotton Research Institute, Hunan Agricultural University. After several years of selection, 15 early-maturing and 15 late-maturing accessions were identified (Table S3). In 2023, these accessions were planted at the Teaching and Research Base of Hunan Agricultural University in Liuyang City, Hunan Province, China (28°18′ N, 113°49′ E). Their maturity was confirmed by recording the bud and flowering stages.

During the squaring stage, leaves from the fourth node below the shoot apex were collected, with three biological replicates per accession. Samples were immediately frozen in liquid nitrogen, transported to the laboratory, and stored at −80 °C for subsequent DNA extraction and quality control.

### 4.2. Genomic DNA Quality Testing and Sample Sequencing

Genomic DNA was extracted from leaf tissues using a modified CTAB method. The integrity of the genomic DNA was assessed by agarose gel electrophoresis, with samples showing a band dispersion of ≤5% considered acceptable. DNA purity was evaluated using UV absorption spectroscopy, and an A_260_/A_280_ ratio between 1.8 and 2.2 was used as the criterion for the absence of substantial protein or RNA contamination. DNA concentration was accurately determined using a Qubit 3.0 fluorometer, with a detection error of <2%. Samples that met the required standards for DNA integrity and purity and had a total DNA amount of at least 1.5 μg were selected for subsequent library preparation.

Genomic DNA libraries were constructed by fragmenting qualified DNA samples to a target size of 350 ± 10 bp using a focused-ultrasonicator (Covaris M220, Covaris, Inc., Woburn, MA, USA). The fragmented DNA was subsequently subjected to end repair and phosphorylation, followed by 3′-end poly(dA) homopolymer extension and ligation of Illumina-compatible sequencing adapters. After adapter ligation, the target fragments were size-selected using AMPure XP beads and enriched by high-fidelity PCR for 15–18 cycles. The qualified libraries were finally subjected to paired-end sequencing (PE150) on an Illumina HiSeq X Ten platform. The sequencing data were evaluated based on sequencing quality, and the obtained clean reads were used for subsequent genomic analysis. The sequencing depth and coverage statistics of each sample are summarized in Table S1.

The raw sequencing reads were subjected to quality control and filtering to generate high-quality clean reads. Reads containing adapter sequences were removed. Paired reads were discarded when the proportion of ambiguous bases (N) in a single read exceeded 10% of the read length. In addition, paired reads were removed when the proportion of low-quality bases (quality score ≤ 5) exceeded 50% of the read length in a single read. The high-quality clean reads were aligned to the upland cotton reference genome (*Gossypium hirsutum* ZJU v2.1 assembly a1) using BWA (version 0.7.17) software with the parameters “mem -t 4 -k 32 -M”. The resulting non-redundant mapped reads were used for subsequent variant calling.

### 4.3. SNP Variant Calling and Annotation

Based on the read alignment results, population SNP variants were identified using SAMTOOLS (version 1.9) and related software. A Bayesian model was applied to detect polymorphic sites among the population. High-quality SNPs were obtained through filtering with the criteria of sequencing depth (DP) ≥3, missing rate ≤0.2, and minor allele frequency (MAF) ≥0.05. The resulting high-quality SNPs were subsequently annotated using ANNOVAR (version 2017Jul16) software.

### 4.4. Detection of Selection Signals

Selection sweep analysis was performed based on the population differentiation index (Fst) and nucleotide diversity (θπ), using a sliding-window size of 100 kb and a step size of 50 kb. Genomic regions with Fst values in the top 1% (Fst > 0.19) and log_2_(θπZ/θπW) values within the upper and lower 1% tails (≤−2.49 or ≥1.44) were defined as regions under candidate selective-sweep regions. Candidate genes within the selected regions were subsequently annotated using the Bedtools intersect algorithm, followed by Gene Ontology (GO) and Kyoto Encyclopedia of Genes and Genomes (KEGG) enrichment analyses.

### 4.5. Candidate Gene Screening and Bioinformatic Analysis

By integrating SNP variants with gene functional annotations, *GH_A11G2894* (*GhGATA9*) was ultimately selected as a priority candidate gene involved in the regulation of early maturity and subjected to subsequent analyses. The protein sequence of *GH_A11G2894* (*GhGATA9*) was first obtained from the CottonFGD database. Its hydrophilicity and hydrophobicity profiles were then predicted using the ProtScale database available on the ExPASy website. The SignalP 5.0 online tool was used to determine whether the GhGATA9 protein contained a signal peptide, while the TMHMM 2.0 online tool was used to predict its transmembrane structure. Conserved functional domains of GhGATA9 were analyzed using the Conserved Domains Search online tool, and its secondary structure was predicted using the SOPMA online tool. The phosphorylation sites of GhGATA9 were analyzed using the NetPhos 3.1 Server. Finally, homologous proteins were searched against the STRING database, and the homologous protein of GhGATA9 in *Arabidopsis thaliana* was identified based on sequence similarity and used to construct a protein–protein interaction network. The URLs of all bioinformatic tools and databases used in this study are provided in Table S4.

### 4.6. Virus-Induced Gene Silencing

To determine the function of the candidate gene in regulating early maturity in cotton, a VIGS vector targeting *GH_A11G2894* (*GhGATA9*) was constructed. The coding sequence of the candidate gene was obtained from the TM-1 v2.1 genome, and primers were designed using Primer 5 (Table S5). A specific fragment of *GhGATA9* was selected based on the coding sequence information of the target gene to specifically target *GhGATA9* and minimize potential non-specific silencing effects. The silencing fragment was cloned into the pCLCrV vector using the restriction enzymes SpeI and AscI and subsequently transformed into *Escherichia coli*. The recombinant pCLCrV-*GhGATA9*, *pCLCrV-PDS*, and empty vector constructs were introduced into *Agrobacterium tumefaciens*. The Agrobacterium suspension was prepared in infiltration buffer containing 10 mM MgCl_2_, 10 mM MES, and 20 μM acetosyringone, and the bacterial suspension was adjusted to an OD600 value of 1.3 before inoculation. The *pCLCrV-PDS* construct was used as the positive control to monitor VIGS efficiency [5]. The upland cotton line 2017-4, which is a late-maturing material, was used for VIGS assays. VIGS inoculation was performed when the two cotyledons were fully expanded. Briefly, the epidermis on the abaxial surface of the cotton cotyledons was gently punctured with the needle of a 1-mL sterile syringe. After removing the needle, the well-mixed inoculum was injected into the cotyledon until the entire cotyledon was filled. After inoculation, the plants were maintained in darkness for 24 h and then transferred to a growth chamber under conditions of 25 °C and a 12-h light/12-h dark photoperiod. For each construct, 20 cotton plants were infiltrated. When the positive-control seedlings (*pCLCrV-PDS*) developed a chlorotic phenotype (approximately 30 days after inoculation), and at the squaring and flowering stages, we collected the third leaf from the top from silenced and control plants, selecting six representative plants for sampling in each group. The relative expression level of the target gene was subsequently determined by quantitative real-time PCR (qRT-PCR). The expression level of *GhGATA9* was quantified using *GhActin* as the internal reference gene, and relative expression levels were calculated using the 2^−ΔΔCt^ method. The squaring date and flowering date of these six plants were recorded for statistical analysis.

## 5. Conclusions

In this study, whole-genome resequencing was performed on 30 upland cotton materials with different maturity types, resulting in the identification of 2,684,227 high-quality SNPs. The integrated Fst–θπ analysis identified 16 significantly selected genomic regions and 80 candidate genes in the early-maturing group. Functional enrichment analysis indicated that these genes were mainly involved in biological processes related to membrane organization, lipid metabolism, and energy metabolism, suggesting that membrane system remodeling and metabolic reprogramming may play important roles in the initiation of early maturity in cotton. Further integration of functional annotations and genetic variant information identified the key transcription factor gene *GhGATA9*, a member of the GATA family, as a priority candidate gene. VIGS functional validation showed that silencing *GhGATA9* significantly delayed squaring and flowering in cotton, indicating that *GhGATA9* may positively regulate the transition from vegetative to reproductive growth. These findings provide new molecular markers and a candidate gene for early-maturity breeding in cotton.

## Figures and Tables

**Table 1:** SNP detection and annotation result statistics.

Category	Number of SNPs
Total	2,684,227
Intergenic	2,360,509
Upstream	75,089
Downstream	63,438
Upstream/Downstream	3939
Splicing	222
Intronic	122,059
Exonic non-synonymous	34,440
Exonic synonymous	20,377
Exonic stop gain	671
Exonic stop loss	215
Exonic unknown	0
Others	3268
ts	1,852,021
tv	832,206
ts/tv	2.225

Total, the total number of detected SNPs; intergenic, variants located in intergenic regions; upstream and downstream, variants located in the upstream and downstream regions of genes, respectively; upstream/downstream, variants located in both upstream and downstream regions; splicing, variants occurring at splice sites; intronic, variants located within introns; exonic non-synonymous, non-synonymous mutations; exonic synonymous, synonymous mutations; exonic stop gain, mutations introducing premature stop codons; exonic stop loss, mutations leading to the loss of stop codons; exonic unknown, exonic variants with unknown functional effects; Ts, transitions; Tv, transversions; Ts/Tv, the ratio of transitions to transversions.

## Data Availability

The raw sequence data reported in this paper have been deposited in the Genome Sequence Archive (Genomics, Proteomics & Bioinformatics 2025) in the National Genomics Data Center (Nucleic Acids Res 2025), China National Center for Bioinformation/Beijing Institute of Genomics, Chinese Academy of Sciences (GSA: CRA032966), which are publicly accessible at https://ngdc.cncb.ac.cn/gsa (accessed on 10 July 2026).

## Supplementary Materials

The following supporting information can be downloaded at: https://www.mdpi.com/article/10.3390/plants15213287/s1, Figure S1: Hydrophilic/hydrophobic analysis of GhGATA9 protein; Figure S2: Prediction of phosphorylation sites and analysis of protein signal peptides of GhGATA9 proteins. A: GhGATA9 protein phosphorylation site prediction; D: GhGATA9 protein signal peptide analysis; Figure S3: Transmembrane domain analysis and secondary structure prediction of GhGATA9 proteins. A: Transmembrane domain analysis of GhGATA9 protein; B: Secondary structure prediction of GhGATA9 protein; Figure S4: Protein–protein interaction networks of Arabidopsis homologs corresponding to GhGATA9. The connections between proteins indicate potential interactions among them; Figure S5: Tissue expression pattern analysis of GhGATA9 in TM-1 transcriptome data. O1~O8 represent the calyx, leaf, petal, pistil, stamen, receptacle, stem, and root, respectively; Table S1: Sequencing Data Statistics; Table S2: Candidate gene list; Table S3: Sample information for 15 early- and 15 late-maturing cotton varieties; Table S4: The bioinformatics websites; Table S5: List of Required Primers.
