# Supplementary material for: Genome-Wide Resequencing Identification of Loci Associated with Early Maturity in Upland Cotton and Functional Validation of GhGATA9
Source: Plants (Basel). 2026 Oct 25;15(21):3287. doi: 10.3390/plants15213287 (PMC13766101; doi:10.3390/plants15213287)
Supplement: Supplementary file 1 [file plants-15-03287-s001.zip › Supplementary Figure.pdf]

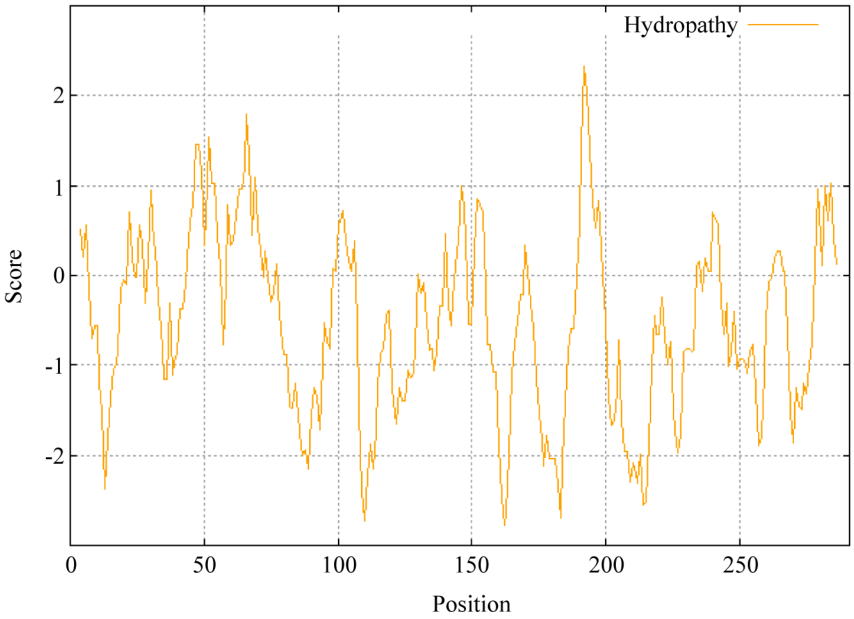

Fig. S1. Hydrophilic/hydrophobic analysis of GhGATA9 protein.

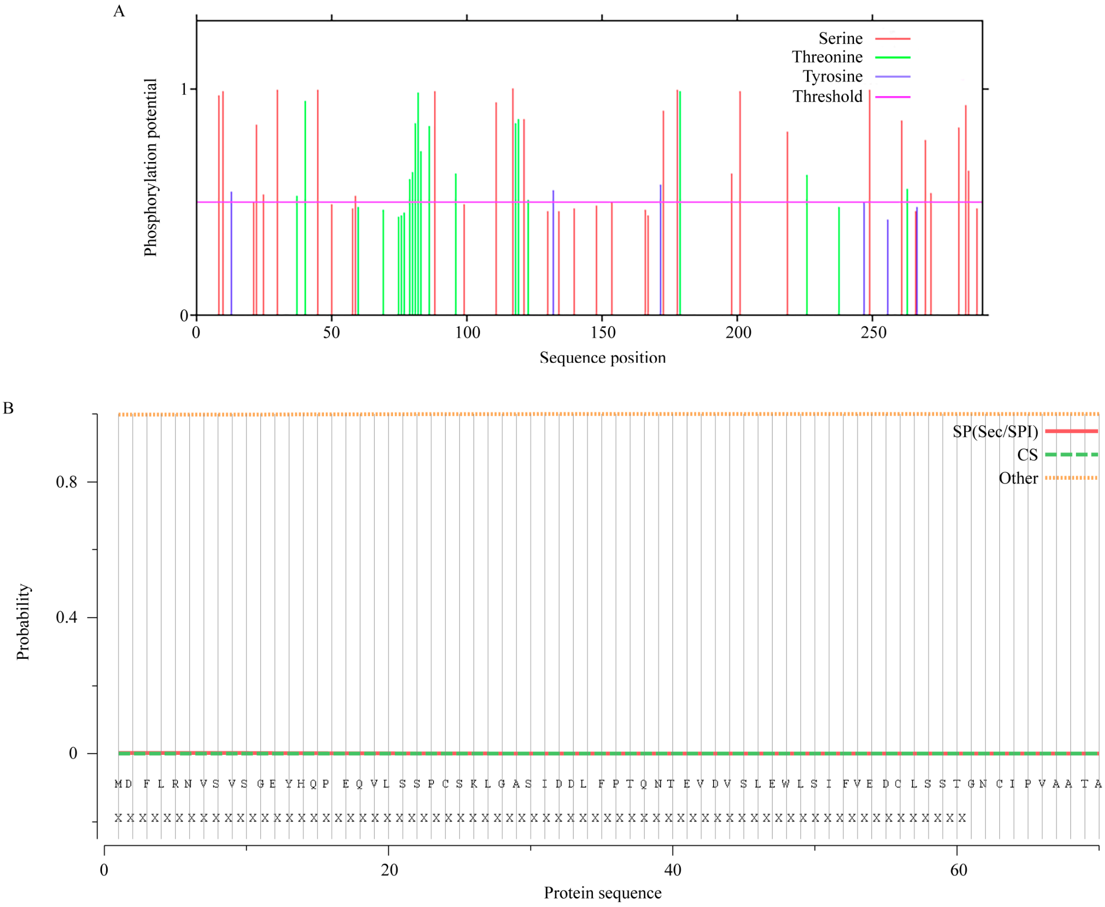

Fig. S2. Prediction of phosphorylation sites and analysis of protein signal peptides of GhGATA9 proteins. A: GhGATA9 protein phosphorylation site prediction; D: GhGATA9 protein signal peptide analysis.

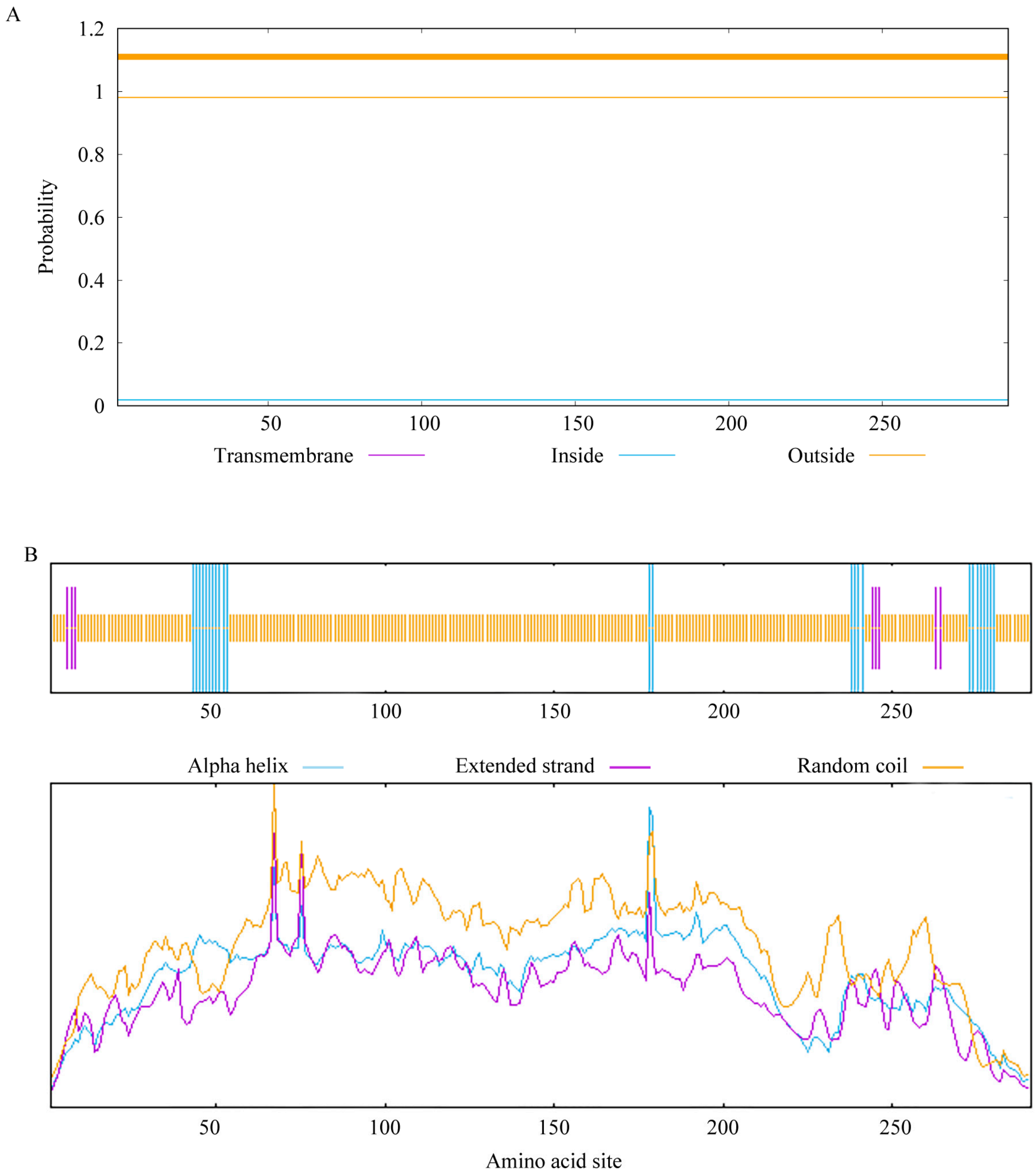

Fig. S3. Transmembrane domain analysis and secondary structure prediction of GhGATA9 proteins. A: Transmembrane domain analysis of GhGATA9 protein; B: Secondary structure prediction of GhGATA9 protein.

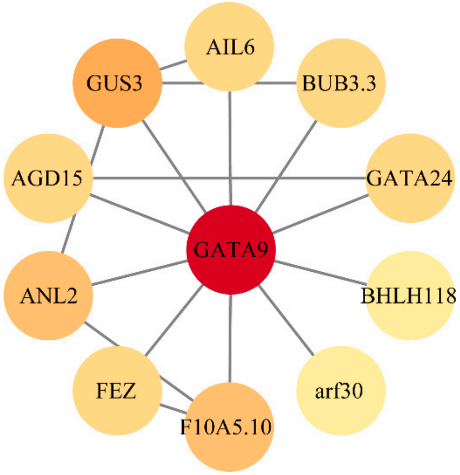

Fig. S4. Protein-protein interaction networks of Arabidopsis homologs corresponding to GhGATA9. The connections between proteins indicate potential interactions among them.

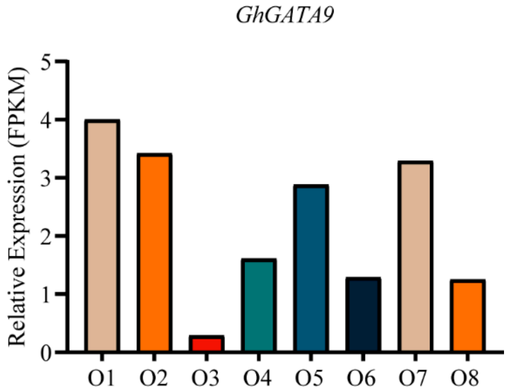

Fig. S5. Tissue expression pattern analysis of GhGATA9 in TM-1 transcriptome data. O1~O8 represent the calyx, leaf, petal, pistil, stamen, receptacle, stem, and root, respectively.
